# Long reads reveal the diversification and dynamics of CRISPR reservoir in microbiomes

**DOI:** 10.1186/s12864-019-5922-8

**Published:** 2019-07-09

**Authors:** Tony J. Lam, Yuzhen Ye

**Affiliations:** School of Informatics, Computing, and Engineering Indiana University, Bloomington, 47408 IN USA

**Keywords:** Synthetic Long-Reads (SLR), Microbiome, CRISPR-Cas system, Spacer gain and loss

## Abstract

**Background:**

Sequencing of microbiomes has accelerated the characterization of the diversity of CRISPR-Cas immune systems. However, the utilization of next generation short read sequences for the characterization of CRISPR-Cas dynamics remains limited due to the repetitive nature of CRISPR arrays. CRISPR arrays are comprised of short spacer segments (derived from invaders’ genomes) interspaced between flanking repeat sequences. The repetitive structure of CRISPR arrays poses a computational challenge for the accurate assembly of CRISPR arrays from short reads. In this paper we evaluate the use of long read sequences for the analysis of CRISPR-Cas system dynamics in microbiomes.

**Results:**

We analyzed a dataset of Illumina’s TruSeq Synthetic Long-Reads (SLR) derived from a gut microbiome. We showed that long reads captured CRISPR spacers at a high degree of redundancy, which highlights the spacer conservation of spacer sharing CRISPR variants, enabling the study of CRISPR array dynamics in ways difficult to achieve though short read sequences. We introduce compressed spacer graphs, a visual abstraction of spacer sharing CRISPR arrays, to provide a simplified view of complex organizational structures present within CRISPR array dynamics. Utilizing compressed spacer graphs, several key defining characteristics of CRISPR-Cas system dynamics were observed including spacer acquisition and loss events, conservation of the trailer end spacers, and CRISPR arrays’ directionality (transcription orientation). Other result highlights include the observation of intense array contraction and expansion events, and reconstruction of a full-length genome for a potential invader (*Faecalibacterium* phage) based on identified spacers.

**Conclusion:**

We demonstrate in an in silico system that long reads provide the necessary context for characterizing the organization of CRISPR arrays in a microbiome, and reveal dynamic and evolutionary features of CRISPR-Cas systems in a microbial population.

## Background

Prokaryotes are constantly engaged in an evolutionary arms-race with mobile genetic elements (MGEs), including phages and plasmids. As invading mobile genetic elements constantly find means to infiltrate their hosts, it becomes unsurprising that prokaryotes have also evolved a multitude of means to defend against such invaders [1–3]. One such defense mechanism is the CRISPR-Cas system, an adaptive sequence-specific immune system present in about half of the bacterial and most of the archaeal genera [4–8]. CRISPR-Cas systems are incredibly diverse, and have a constantly changing classification scheme owing to the constant discovery of new CRISPR-Cas system subtypes [2, 9, 10]. The diversity of CRISPR-Cas systems have been suggested to be attributed to the evolutionary arms-race between prokaryotes and their invaders [11–13]. Similarly to the evolutionary diversity of CRISPR-Cas systems, invaders such as phages have also been observed to evolve in tandem to evade host defense mechanisms, such as anti-CRISPR genes which are among some of the recently discovered mechanisms [1, 2, 14–17].

CRISPR arrays are comprised of short DNA segments, known as spacers provide a cornerstone to CRISPR-Cas derived adaptive immunity. Spacers, which were originally segments of the invaders’ genomes, retain the memory of past immunological encounters and are primarily acquired as a result of Cas protein complex mediated acquisition [2]. Newly acquired spacers are typically integrated towards the leader ends of arrays [18]. Additionally, leader sequences usually found upstream of CRISPR arrays were attributed to the efficiency of CRISPR-Cas derived immune response [19]. However, several studies have also suggested that spacer acquisition remains possible through several alternative means such as homologous recombination [18, 20, 21], and ectopic spacer integration where spacers are inserted into middle of arrays as a result of leader sequence mutations [19, 22].

While mechanisms of spacer acquisition have been widely studied, direct evidence has yet to emerge to suggest the existence of a dedicated biological mechanism for the systematic deletion of CRISPR spacers. Several observations have promoted hypotheses to explain the modes in which spacers could be lost within CRISPR arrays. Just as how homologous recombination can enable the acquisition of spacers, homologous recombination has also been shown to provide a means for spacer deletion [4, 14, 20, 23–26]. Additionally, as even some of the largest of CRISPRs have been shown to contribute only to no more than ∼ 1*%* of the genomes [27], it is hypothesized that there remains a biological function effectively suppressing the indefinite growth of CRISPRs. The maintenance of CRISPR array size is thought be related to the upkeep of CRISPR defense efficacy, and fitness cost optimization [14, 23, 26, 28, 29]. Furthermore, it has also been found that DNA polymerase slippage during replication may induce low levels of CRISPR loci deletion variants [20, 30, 31].

Until recently, much of the work surrounding the analysis of CRISPR arrays, and more broadly the CRISPR loci, have originated from the analysis of datasets generated from next-generation sequencing. Next-generation sequencing has enabled the expansion and availability of sequencing technology, providing the vehicle which helped expand our fundamental understanding of biology and biological processes. However, as with all technologies, next-generation sequencing is not without its own drawbacks. To note, one of the major technical challenges of analysis regarding short reads stems from repetitive sequences [32]. Repetitive regions in CRISPR arrays pose computational challenges for assemblers where the assembly of repeat containing reads can result in erroneously collapsed reads, chimeric contigs, and fragmented assemblies [32–34]. Despite the recent developments of computational tools, such as metaSPAdes for metagenome assembly [35], challenges surrounding the accurate assembly of repetitive regions using short reads still remain.

Considering the advancements in sequencing technology, third generation long read sequencing techniques have provided a means to address much of the current concerns surrounding next-generation sequencing such as haplotype phasing, structural variant detection, and short reads assembly [36]. Among the third generation sequencing technologies are Nanopore sequencing, PacBio’s SMRT sequencing, 10 × Genomic’s Chromium technology, and Illumina’s TruSeq Synthetic Long-Read (SLR). Long read sequencing has been shown effective in resolving regions of the genome where short reads were unable to map uniquely, such as repetitive regions [37]. The ability to provide an accurate mapping of repetitive regions has proven effective in uncovering large segments of genomes previously inaccurately assembled [38–42].

Here we investigate the utilization of long read sequences derived from a gut microbiome [43] for the application of studying CRISPR-Cas system dynamics, focusing on acquisition and loss of spacers, in the underlying microbial community. Using the computational tool that we have previously developed for the characterization of CRISPR-Cas systems [44], combined with new tools we developed for comparing and visualizing the CRISPR arrays, we study the dynamics of CRISPR arrays using long reads. One of such tools is compressed spacer graphs, a visual abstraction of spacer sharing CRISPR arrays, used to construct a simplified representation of complex organizational structures present within CRISPR array dynamics by simplifying common shared features and emphasizing those that vary. While the study of CRISPR array dynamics are not unique, previous studies have been restricted to studying microbiome samples through time series [18]. Additionally, previous studies of CRISPR array dynamics were often restricted to carefully curated single species experiments, limiting the scope of the study to single species [14, 20, 23, 26, 28, 45]. Our initial findings suggest that long reads provide a greater depth of spacer *redundancy* (multiple observations of the same CRISPR spacer sequence within a given sample), enabling the analysis of dynamics of CRISPR arrays in a microbial community using single time point microbiome data.

## Results

We applied our tools to characterize CRISPR arrays in a gut microbiome, which was sequenced using both short (Illumina) and long sequencing technologies (SLR) [43]. Comparison of the results showed that long reads contain necessary genomic contexts for analyzing CRISPR organizations, owing to the facts that CRISPR repeats and spacers are typically short (less than 50 bps) and a CRISPR array typically contains a few or up to a few dozens of spacer-repeat units. We built spacer graphs for groups of CRISPR arrays that share spacers. The spacer graphs revealed a broad spectrum of CRISPR array organization diversity in the gut microbiome. In addition, by examining the spacer graphs, we were able to identify important dynamic and evolutionary features of CRISPR arrays in the gut microbiome.

### Long reads retain the redundancy of CRISPR spacers critical for CRISPR organization analysis

We first compared CRISPR arrays predicted from both the long-reads and short-reads datasets of the gut microbiome. CRISPR arrays from long-reads were predicted using entire reads, whereas CRISPR arrays predicted from short reads were predicted from assembled contigs as short reads themselves are too short to provide meaningful information regarding the arrangement of spacers in CRISPR arrays. Spacer sequences were extracted from the identified CRISPR arrays and were labeled by clustering spacers at 90% sequence identity (see “Methods”). The resulting ratio of spacer clusters to number of predicted spacers indicates the redundancy of spacers found within the sample.

Table 1 summarizes the comparison. A total of 1211 and 2034 spacers were predicted from the contigs assembled by MEGAHIT [46] and metaSPAdes [35], respectively. These spacers were clustered into 1195 and 2015 spacer clusters, respectively. The difference in the number of spacers predicted from short read contigs compared to long reads suggests that the number of spacers predicted from short reads are dependent on the assembly method used. The discrepancy observed between assembly methods are most likely attributed to the complications of assembling repetitive regions in CRISPR arrays. Previous evaluations of metagenomic assemblers have shown that MEGAHIT assemblies have fewer structural errors compared to metaSPAdes, while metaSPAdes contains fewer under/over collapsed repeats when compared with MEGAHIT contigs [47]. Nevertheless, both assembly methods yielded similar spacer redundancy scores of ∼ 1.01. The redundancy scores indicate that spacers predicted in assemblies of short reads, on average, had only a single copy and were unique in comparison to other predicted spacers. The low redundancy of predicted spacers found in short reads assemblies makes the analysis of spacer organization and their dynamics nearly impossible. In comparison, CRISPRs predicted through long reads yielded a total of 51,416 spacers, which clustered into 5685 spacer clusters. Long read CRISPR spacers yielded a redundancy score of ∼ 9.04, which indicates on average each spacer found within the sample appears approximately 9 separate times. The observed redundancy of spacers remains critical for revealing the potential diversity of CRISPR array organization, and is important for any potential analysis regarding the loss and gain of spacers of CRISPRs within bacterial communities.

**Table 1:** Comparison of CRISPR characterization using long reads versus short reads (assembled) of the gut microbiome

Dataset	Bps	Assembler	# of spacer	# spacer cluster	redundancy
Long read (SRR2822456)	8.4Gb	N/A	51416	5685	9.04
Short read (SRR2822459)	7.6Gb	MEGAHIT	1211	1195	1.01
		metaSPAdes	2034	2015	1.01

Note: two different assemblers (MEGAHIT and metaSPAdes) were employed to assemble the short reads and spacer identification results were shown in the table; the redundancy of spacers was measured as the #of spacers / # of spacer clusters

To ensure that the CRISPR array variants and dynamics observed in the gut microbiome are not an artifact of the sequencing technology, we analyzed a separate mock microbiome derived from a synthetic community of 20 known bacterial species sequenced using the same long read sequencing technology (i.e., TruSeq SLR) [43]. A total of 5 groups of reads containing spacer-sharing CRISPR arrays were identified from the mock dataset, each group containing at least 10 reads. As expected, we observed no changes to the CRISPR array organization in all these groups. For example, the largest group has 493 reads, among which, 428 reads contain the same, complete array with 12 spacers, and the rest contain shorter arrays (because the reads are fragmented). The smallest group has 10 reads, containing the longest CRISPR array (with 25 spacers) among the five groups. Again, no spacer reorganization was observed among these arrays. In summary, the mock dataset did not have variations in their CRISPR organization, confirming that no artificial variations of the CRISPR arrays were produced by the SLR approach. It also suggests that the results we observed for the real gut microbiome dataset were unlikely to be artifacts resulting from the sequencing technology. We note that all the results concerning the diversity of the CRISPR arrays in this paper are based on the gut microbiome dataset.

### Spacer graphs provide visual summaries and are useful for studying the patterns of CRISPR spacer acquisition

Observations of high spacer redundancy within long read sequences in the gut microbiome (Table 1) suggest that many of the CRISPR arrays predicted within long reads are spacer sharing CRISPRs. Using the greedy algorithm we developed (see “Methods”), we clustered the CRISPR arrays into 252 groups, among which 105 are singletons and 41 each contain at least 10 spacer-sharing CRISPR arrays. We focused on the groups each with at least 10 arrays and built compressed spacer graphs for them. Given the depth of redundancy of spacers found in long reads, compressed spacer graphs have shown the ability to capture CRISPR spacer dynamics involving the acquisition, retention, and loss of spacers.

**Fig. 1:**
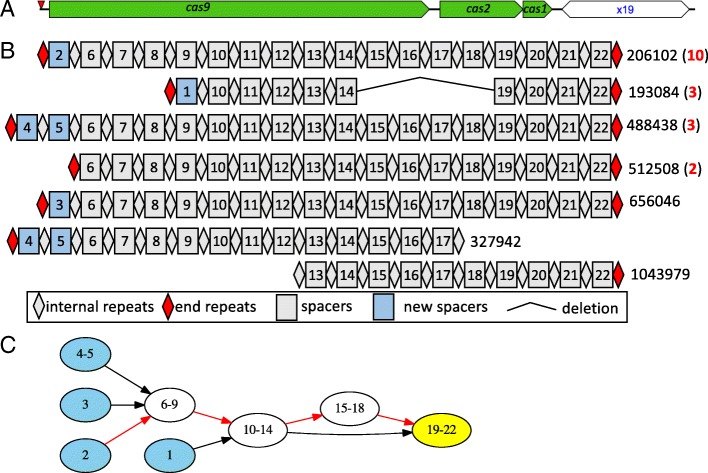
A group of type II CRISPR arrays captured in long reads containing shared CRISPR spacers. **a** Representative CRISPR-Cas system characterized from a single long read (SRR2822456.206102, reverse complement). Green arrows represent *cas* genes, open hexagon represents a CRISPR array containing 19 repeats, and the red arrow denotes a putative anti-repeat that may be part of the tracrRNA [62]. **b** Representative organization of spacer-sharing CRISPR arrays. Spacers are each represented as a square with a unique spacer ID, whereas diamonds represent the repeats. The number of reads found to contain a CRISPR array with the same organization of spacers are denoted in red, right of the representative sequence. Complete CRISPR arrays, where ends of arrays are not fragmented or truncated as a result of sequencing, are denoted by a red diamond at the end of CRISPR arrays; fragmented ends of arrays remain as a gray diamond. **c** Compressed spacer graph constructed from CRISPRs sharing spacers. Uninterrupted blocks of repeat-spacer units are represented as a single node. Directed edges between nodes indicate the ordering of spacers found in a CRISPR array, where the path consisting of red edges represent the most frequent spacer-repeat organization observed in the group of CRISPRs. In the compressed spacer graph, source nodes (without incoming edges) are highlighted in blue, and they are likely to contain newly gained spacers; the sink node (without outgoing edges), which contains the trailer end spacer, is highlighted in yellow

We used spacer sharing CRISPR arrays of a type II CRISPR-Cas system to demonstrate compressed spacer graph and its utilities (Fig. 1). A read (SRR2822456.2206102) was found to contain an intact type II CRISPR-Cas system with *cas* genes next to the identified CRISPR array (Fig. 1a). Using the CRISPR arrays that share spacers, an alignment of the CRISPR spacers (Fig. 1b) shows that while CRISPR arrays maintain a common predominant structure, various acquisition and loss events were observable between arrays. As identical spacers, and those computationally similar, were grouped into the same node, the organizational structure of the CRISPR arrays becomes more apparent. The compressed spacer graph is a directed graph, with directed edges representing the sequential ordering of the spacers found in the arrays. The most frequently observed path in the compressed spacer graph, depicted with red edges, begins with spacer 2 and continues from spacer 6 to 22. Alternative paths to the dominant path show the diversity of CRISPR arrays, and highlight the acquisition and loss events within the cohort of arrays. Collectively, the compressed spacer graph (Fig. 1c) suggests that spacers 1-5 are likely to be the more recently acquired spacers, while the remaining spacers 6-22 represent the core structure of the CRISPR arrays. We also note that several reads (3) contain CRISPR arrays with a loss of four spacers (14-17), as shown in Fig. 1b, which is shown as a separating node providing an alternative route in the graph from node (10-14) to node (19-22) in Fig. 1c. A similarity search using the representative read belonging to this group (SRR2822456.206102) as the query against NCBI nucleotide database revealed that this CRISPR-Cas system is most similar to the type II CRISPR-Cas system in the *Parabacteroides sp.* CT06 genome (CP022754.1, which has both a type I and type II CRISPR-Cas system), with 97% sequence similarity covering the whole region except for the CRISPR spacers: the two arrays (one identified from read SRR2822456.206102 and the other one identified from CP022754.1) shared only one spacer in the distal end (shown on the right in Fig. 1c), i.e., the oldest spacer with sequence of TGCAATCGCATTGAACCAAAACGCAGAGAA.

In Fig. 1c, the direction of the edges and also the layout of the compressed spacer graph were oriented such that the CRISPR ends with active spacer acquisitions are shown on the left, whereas the trailer ends are shown on the right. Compressed spacer graphs not only provide visualization of the CRISPR arrays, but also useful information for inference. Compressed spacer graphs in some instances are able to provide information regarding the directionality (i.e., transcription orientation) of active CRISPR arrays: CRISPR arrays are transcribed and processed to generate small CRISPR RNAs (crRNAs), guiding the targeted immunity of the systems. In our analyses, we first determine the orientation of the CRISPR array (from left to right in the figures) using our own analysis based on repeat degeneracy associated with the distant end of the arrays, combined with the inspection of the compressed spacer graphs. Notably, the orientation of CRISPR arrays belonging to those in Fig. 1 are opposite of CRISPRDetect’s predicted orientation (CRISPRDetect [48] provides a high confident prediction of orientation supported by multiple lines of evidence, including secondary structural analysis prediction, array degeneracy analysis prediction, and AT richness analysis in flanks). This suggests that predicting the directionality of the CRISPR arrays based on sequential composition is still a challenging problem. Surprisingly, the spacer graph representation of spacer sharing type II CRISPR-Cas systems in Fig. 1 reveals variance of proximal end spacers and conservation of distal end spacers, which together suggest that proximal end spacers were more recently acquired, providing inference to the directionality of the CRISPR arrays. Inspired by this example, we inferred the CRISPR orientations by inspecting their corresponding compressed spacer graphs, for all the CRISPR array groups each representing at least 10 arrays, in combination with our own analyses of repeat degeneracy and CRISPRDirect prediction results. The results and visualization of all compressed spacer graphs generated in this study are available at our supplementary website (http://omics.informatics.indiana.edu/CRISPRone/long).

### Compressed spacer graphs reveal a broad spectrum of CRISPR array organization diversity

Compressed spacer graphs defined from CRISPRs predicted from the gut microbiome dataset exemplified a broad spectrum of CRISPR organizational structure and complexity even for a single population of microbial organisms. The absence of branching within observed compressed spacer graphs indicates that all CRISPR arrays used to construct the compressed spacer graph shared identical organization of spacers, whereas compressed spacer graphs with numerous branches had large amounts of spacer sharing CRISPR array variants which shared some but not all spacers. See the different compressed spacer graphs in our supplementary website (http://omics.informatics.indiana.edu/CRISPRone/long): examples of simple graphs (without branching structures) include cluster 6 (consisting of 132 arrays), cluster 13 (consisting of arrays) and cluster 20 (consisting of 29 arrays); examples of complex graphs include clusters 1-5, and cluster 9 (with 80 arrays). These contrasting examples highlight both the active and stagnant nature possible of CRISPR-Cas systems.

**Fig. 2:**
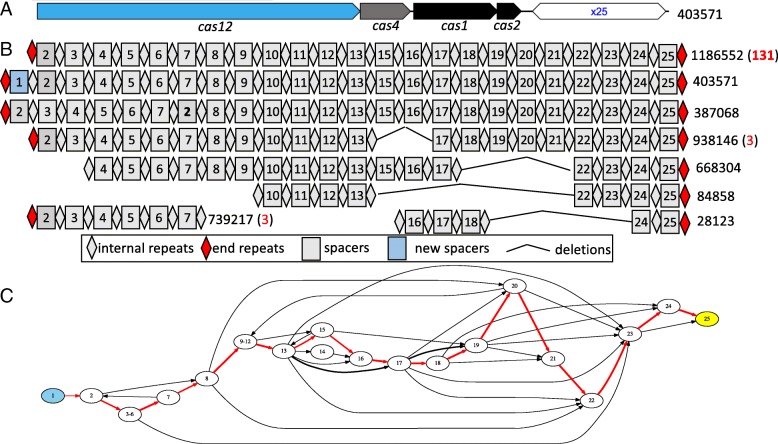
Spacer-sharing in CRISPR arrays associated with a type V CRISPR-Cas system. **a** shows a representative of this CRISPR-Cas system predicted from long read (SRR2822456.403571), with both the array and adjacent *cas* genes; **b** shows representative organizations of the spacers involved in these arrays. **c** shows the compressed spacer graph constructed from the CRISPR arrays. See Fig. 1 caption for the notations

**Fig. 3:**
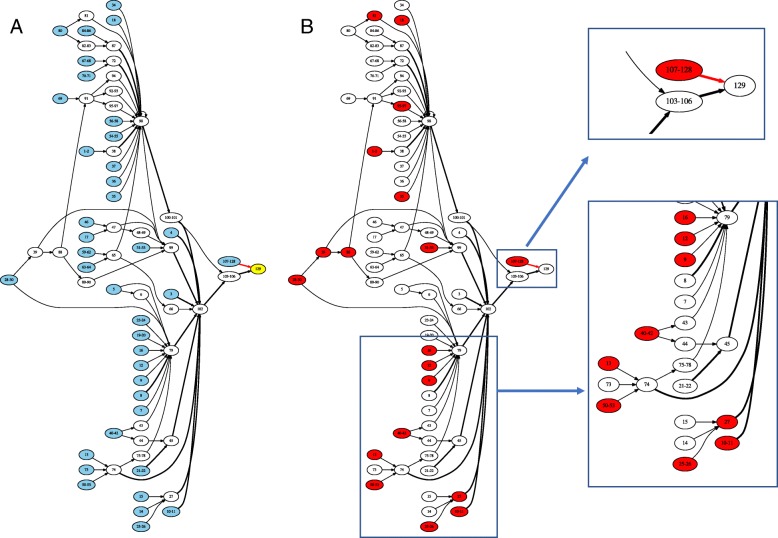
A compressed spacer graph representing diversification of CRISPR arrays via extensive spacer gains. In (**a**), the nodes are colored according to their topological property with source nodes (which are likely more recently gained spacers than the rest) shown in blue and the sink node (the conserved spacer in the trailer end) in yellow. In (**b**), the nodes representing spacers with matching co-occurring protospacers (found in the same microbiome) are shown in red

In addition to revealing a varying range of observable states among spacer sharing CRISPRs, compressed spacer graphs also reveal other integral aspects of CRISPR dynamics such as the contraction and expansion of CRISPR arrays. Here we showcase two compressed spacer graphs which provide snapshots of periods of intense expansion and contraction of CRISPRs induced by the rapid acquisition and loss of spacers (Figs. 2 and 3).

We first exemplify a cluster of spacer sharing type V CRISPR-Cas systems exhibiting pervasive CRISPR contraction through various spacer loss events (Fig. 2). Type V CRISPR-Cas systems are among some of the more recently characterized CRISPR-Cas systems [10], and contain the hallmark *cas12* gene (formerly *cpf1*). Figure 2a illustrates a type V CRISPR-Cas system identified in long read (ID:403571). The *cas12* gene identified in this read is similar to other *cas12* genes collected in the NCBI protein database, but is most similar to those identified from *Lachnospiraceae* (Strain: ND2006, sequence ID: WP_051666128.1), however sharing only ∼ 47*%* amino acid sequence identity. The rapid spacer loss exemplified in this example is observed through the multi-spacer gaps in alignment to the reference CRISPR array (Fig. 2b). These segmental loss of portions of the CRISPR array result in long alternative branches in the compressed spacer graph (Fig. 2c), and may be a result of recombination events.

In addition to CRISPR contractions, compressed spacer graphs are able to capture periods of intense CRISPR expansion characterized by the massive gains of spacers. CRISPR expansion is exemplified in Fig. 3, which illustrates a compressed spacer graph constructed from 173 arrays involved with a type I CRISPR-Cas system. All arrays within the compressed spacer graph shown in Fig. 3 share the same CRISPR repeat, including the CRISPR arrays containing spacers 107-129, which share only a single spacer (spacer 129) with other arrays within the graph. Figure 3a shows the rapid expansion of identified CRISPR arrays, with leader end spacers identified as likely new spacers denoted in blue. Extreme diversity is exemplified in this compressed spacer graph as a substantial number of (131) unique spacers were identified from the collection of CRISPRs. We note that while the compressed spacer graph is comprised of a large cohort of unique spacers, the overall structure of the compressed spacer graph is maintained by a set of approximately a dozen core spacers commonly shared between the CRISPRs.

In both cases of intensive expansion and contraction of CRISPR arrays, compressed spacer graphs were able to simplify the underlying features of identified CRISPR arrays. While we were able to observe the extreme variations between spacer sharing CRISPR arrays, we hypothesize that not all CRISPR variants will persist through the population as selective pressures will enrich for variants with greater evolutionary advantage. Additionally, few compressed spacer graphs were observed to have as much CRISPR variants as in Figs. 2 and 3. As such, we hypothesize that while there exist periods of rapid spacer gain and loss, most of the resulting CRISPR variants do not persist within the population, otherwise the observable branching within other compressed spacer graphs would be more persistent.

Of notable interest, spacer loss was not observed at the trailer end of identified CRISPR arrays. We observed high conservation of the trailer end spacer across majority of the CRISPR arrays used to construct compressed spacer graphs. In Fig. 2, among the 303 reads used to construct the compressed spacer graph, 263 of the reads were predicted to have spacer number 25 as the trailer end spacer. Figure 3 similarly exhibited high conservation of the trailer end spacer in majority of the reads where 173 reads were used to construct the compressed spacer graph, and 169 of those reads were predicted to have spacer number 129 as the trailer end spacer. As the trailer end spacers are highly conserved across spacer sharing CRISPR variants, we refer to these trailer end spacers as ‘anchor’ spacers. These anchor spacers are the sink nodes in directed compressed spacer graphs, and are illustrated as yellow nodes. Our observations of ‘anchor’ spacers are consistent with previous studies which have also found conservation of trailer-end spacers using temporal data of single species [18, 26].

### Caught in action: co-existence of the defense systems and invaders in microbial communities

An integral part of studying CRISPR-Cas system community dynamics relies on the identification of spacer targets and protospacer sources. Each spacer sequence within a CRISPR array is acquired from a fragment of foreign genetic material known as a protospacer; this incorporation of foreign genetic material characterizes the acquired immunological memory commonly associated with CRISPR-Cas systems [5]. Analyzing spacer sequences of identified CRISPR-Cas systems, we search for potential protospacer sequences within the same microbiome sample used to predict the CRISPR-Cas systems. The ability to identify intra-sample spacer targets provides the opportunity to identify active MGE targets of spacers rather than inference through sequence similarity of genome databases. Searching against intra-sample targets, we were able to identify a significant portion of potential protospacer targets. We exemplify the abundance of intra-sample protospacer matches in Fig. 3b, where we highlight spacers with matching putative protospacers. The identification of potential intra-sample protospacers suggests practical application of long read sequencing for observing the co-existence of invader and defenders within the same community.

**Fig. 4:**
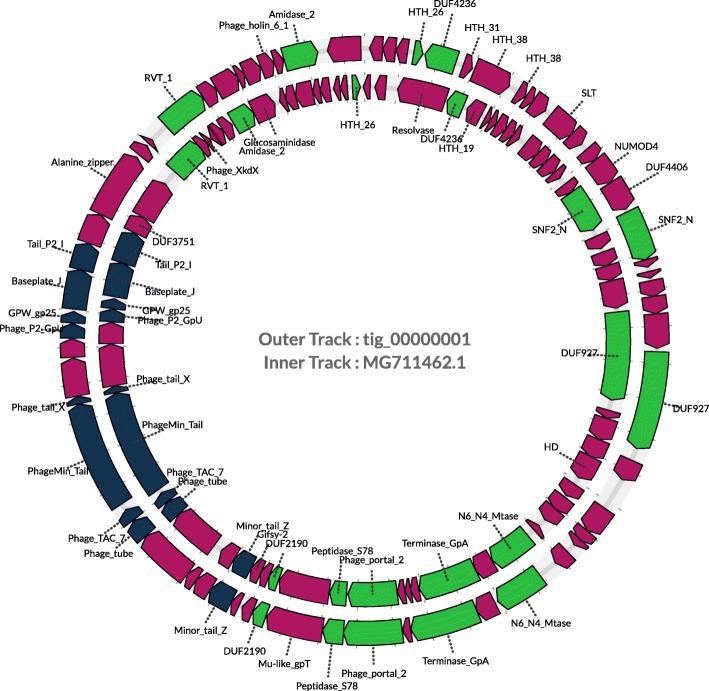
Comparison of the phage genome we assembled and *Faecalibacterium* phage FP_Epona genome (MG711462.1). Genes were predicted using FragGeneScan [32] and annotated using Prokka [58], and are shown as arrows in the figure. We also used similarity search to assign functions from MG711462.1 to tig0000001. Genes shared between both genomes are colored in green, while genes encoding phage tail proteins are shown in blue, and all other putative genes are colored in red

Further exploring the practical application of identified CRISPR spacers, we identified non-CRISPR associated reads which matched based on sequence similarity to predicted spacers likely sampled from invaders containing protospacers. Using these reads as the input, we applied Canu [49] to assemble longer contigs that represent putative invaders. In total we were able to derive 61 contigs, of which, 19 were larger than 20 Kbps. Among these 19 contigs, 12 each contain at least one gene encoding for phage-associated proteins (including phage structural proteins and primase) and one contains a gene encoding for plasmid-associated protein, indicating their potential sources as plasmid, phage, or prophage-containing genomes. In particular, the longest contig (tig00000001) was found to contain overlapping ends allowing for the circularization of the contig (Fig. 4). The derived circular genome was 48843 bp in length, and found most similar with *Faecalibacterium* phage FP_Epona genome (MG711462.1). Figure 4 illustrates the overall similarity of the two genomes with contrasting differences. We note that the putative phage genome was assembled using long reads from the gut microbiome dataset, demonstrating long read sequencing’s ability to capture the co-existence of both invaders and hosts within the microbiome.

## Discussion

Features of next-generation sequencing such as the inaccurate assembly of repetitive regions pose challenging hurdles and limit the use of short read sequences to properly study CRISPR-Cas system dynamics. Here we show that long read sequences are able to provide greater context to CRISPR arrays identified within a microbiome. Comparing both short read and long read sequences from the same sample source, we show that long read sequences contain greater spacer redundancy, owing to the greater abundance of spacer sharing CRISPR variants found within long read samples. The contrasting differences of spacer redundancy between short and long reads suggest that short read sequences (and their assemblies) may not provide the necessary context to study the dynamics between CRISPR-Cas systems and their targets. Evaluating CRISPR arrays predicted through long read sequences, we introduce compressed spacer graphs to provide a simplified abstraction of spacer sharing CRISPR organization. Previous studies often focus on the comparison of spacers (without considering the arrangement of the spacers in the arrays) [32, 50], while other studies use pileups of CRISPR arrays (in which spacers are aligned) to show the commonality and differences of the CRISPR array organization. While the pileup alignments of identified CRISPR arrays are useful in providing information regarding the conservation of spacers between different arrays, it remains difficult to compare large sets of CRISPRs to reveal the underlying structures. Taking CRISPR array pileups a step further, we represent the alignment of CRISPR arrays as a graphical model and collapse non-branching nodes to simplify the relationship between CRISPR variants. Using compressed spacer graphs, we were able to observe various aspects of CRISPR array dynamics such as compression and expansion events between CRISPR array variants. While compressed spacer graphs are able to highlight subtle features of spacer graphs, they also cause the loss of some notable features in comparison to spacer array pileups. Features such as spacer abundance, and array length information are inevitably lost through the clustering and generalization of this method. Nevertheless, spacer graphs offer an complementary method for the visualization and representation of spacer sharing CRISPR arrays, and offer a tractable method of analyzing large spacer sharing CRISPR communities. The simplified abstraction of compressed spacer graphs also allows for the easy characterization of core CRISPR structures, and uncovers notable features such as ‘anchor’ spacers.

Interestingly, for arrays with high variance, compressed spacer graphs were able to provide subjective information in regards to the directionality of the arrays. As spacers are commonly acquired at the leader ends of arrays, compressed spacer graphs provided inferred direction to observed arrays. Directionality of CRISPR arrays remain difficult to correctly characterize. Various CRISPR prediction tools, including CRISPRstrand [51] and CRISPRDirect [52], have provided inference to the directionailty of CRISPR arrays using features such as flanking AT content, and repeat degeneracy. Another attempt to infer the transcription direction of CRISPR arrays also includes the use of metatranscriptomic data as added features for prediction[53]. However, these methods cannot provide high-confidence predictions for the CRISPR arrays if they lack some of the mentioned important features for prediction. Here we show the variance of spacer sharing arrays provides a high confidence marker for the directionality of arrays, and is easily visualized through compressed spacer graphs. We expect that these CRISPRs can supplement the limited collection of CRISPR arrays with verified transcription direction for training better models for prediction.

Previous studies studying CRISPR-Cas dynamics have relied on studying genomic databases, carefully curated single-species experimental designs, or the collection of multiple samples over an extended period time [14, 20, 23, 26, 28, 45]. In contrast, the spacer redundancy of long read sequencing enables the ability to capture CRISPR community dynamics which were previously difficult to achieve using short reads. In this study, we used the TruSeq SLRs, synthetic long reads produced using a combination of a specialized library prep method for strand tagging and assembly for the construction of long reads [43]. We acknowledge that while SLRs are able to capture more information than short reads, they still carry some of the issues associated with short read sequencing such as GC bias [54]. One advantage of using SLRs is that they are more accurate than those of single molecule long read sequencing technologies such as PacBio and Oxford Nanopore. If the long reads contain high errors (e.g., 10% or higher), it would become difficult to determine if two spacer sequences are different because they are different spacers, or they are the same spacer but full of errors, limiting the applications of long reads with low errors for the studies of CRISPR array dynamics. Nevertheless, we believe that as the accuracy of long reads technologies keeps improving, tools we have developed will be able to generalize. Meanwhile, we will explore new approaches of characterizing spacer sequences with high errors, again by utilizing the redundancy of spacers, assuming sequencing errors are random so can be canceled out.

As we have shown, CRISPRs predicted through long read sequencing coupled with compressed spacer graphs were able to reveal similar patterns of conserved trailer end spacers as previous studies [18, 26], but provided the added advantage of achieving the same observations though a single time point. Lopez-Sanchez et. al’s study involved a subset of *Streptococcus agalactiae* strains isolated from various sources, and Weinberger et. al’s study explored the evolutionary dynamics of CRISPRs and their targets through temporal metagenomic datasets of acid mine drainage systems spanning over 6 years. Both studies have found similar features of conserved trailer end spacers. However, unlike previous studies, our observations are based on a single “snapshot” of CRISPR-Cas systems of a microbial community, reflecting the CRISPR diversity and organization of a bacterial population at a given time. While this involved single time point microbiome data, we do not exclude the potential of applying our methods utilizing temporal data, but rather we wish to highlight the resolution in which our methods have been able to capture, even using just a snapshot of a microbial community.

Here we demonstrated the power of using long sequencing techniques in studying the organization of CRISPR arrays. We anticipate that long reads will be key to studying other types of hypervariable regions in microbial communities. Currently, applications of long read sequencing to microbiome study are still scarce. However, considering the rapid advances of sequencing technologies, we anticipate there will be no shortage of such studies in near future.

## Conclusions

Using a single TruSeq dataset of gut microbiome and tools we have developed, we were able to reveal the CRISPR array organizations for dozens of CRISPR-Cas systems belonging to various subtypes including type V, showing the power of using long reads for characterizing the dynamics of genetic elements involving repetitive regions such as the CRISPR arrays in a microbial community. We anticipate that our approaches can be applied to other long sequencing reads (such as the 10 × genomics) of microbiome.

## Methods

### Identification of CRISPR arrays and *cas* genes

CRISPR-Cas systems were computationally predicted from SLRs using CRISPRone [44]. Utilizing CRISPRone results, the orientation of CRISPR arrays were inferred through the analysis of the degeneracy of CRISPR repeats within the putative arrays. CRISPRDetect [48] was also used to provide additional analysis in regards to the orientation of putative CRISPR arrays.

Spacer sequences were extracted from the identified CRISPR arrays and were then clustered at 90% sequence identity (by cd-hit-est [55]). We used 90% identity to allow a small number of sequencing errors and real mutations found in spacers. Spacer sequences in the same cluster were considered as the *same* spacer such that the CRISPR arrays could be represented as sequences of spacer identities.

### Clustering of spacer sharing CRISPR arrays

CRISPR arrays represented as sequences of spacers were then compared and clustered based on the sharing of spacers. We developed a greedy approach for the clustering of CRISPR arrays. The greedy approach first selects a reference CRISPR array with the largest number of spacers which has yet to be recruited into a cluster. It then assesses CRISPR arrays which have yet to be clustered with existing clusters for shared spacers; the CRISPR array is added to a cluster if it shares at least one spacer with a clustered CRISPR, else it will be used as the reference for a new cluster. This procedure is repeated until all CRISPR arrays are grouped into clusters.

### Construction of compressed spacer graphs

**Fig. 5:**
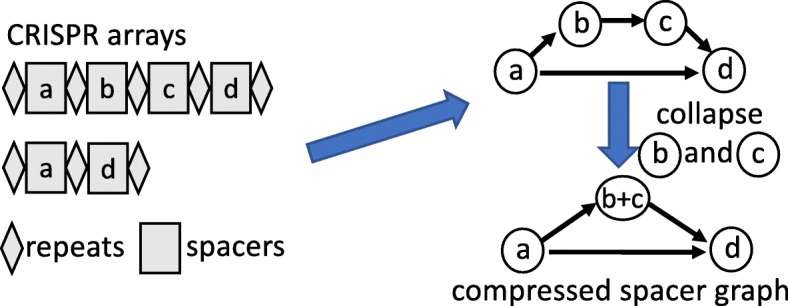
A schematic diagram to demonstrate how to generate a compressed spacer graph from spacer-sharing CRISPR arrays

Given a group of spacer sharing CRISPR arrays, a graph was built to represent the “wiring” of spacers between separate CRISPR arrays. Directed graphs are constructed with nodes representing spacers, and edges represent the sequential linkage between those spacers. We further simplify the spacer graph by collapsing a node with its neighboring node if both nodes share an “in-degree” and “out-degree” equal to or less than one. For example, a CRISPR array containing four consecutively ordered spacers (a, b, c, and d) results in a spacer graph with four nodes with three directed edges: (1) a to b, (2) b to c, and (3) c to d. Adding a second CRISPR array containing two consecutive spacers (a, and d) to the existing spacer graph will produce an edge from a to d (see Fig. 5). As nodes b and c both have an “in-degree” equal to one and “out-degree” equal to one, we collapse nodes b and c. The resulting graph will consist of three nodes and three edges: (1) a to [b-c], (2) [b-c] to d, and (3) a to d. We refer to our graphs as **compressed spacer graphs** to distinguish itself from spacer graphs used in [43, 56] which were used for different purposes.

All observed CRISPR arrays can be represented as a path in the compressed spacer graph. Compressed spacer graphs provide a visual abstraction of spacer sharing CRISPR arrays, and also provide a simplified view of complex organizational relations between spacer sharing CRISPR arrays, simplifying shared features while highlighting the differences between arrays. Additionally, compressed spacer graphs also remain useful in revealing patterns which govern the evolution of CRISPR arrays such as, but not limited to, the acquisition and loss of spacers, and the directionality of CRISPR arrays.

### Intra-sample invader identification

Spacers were extracted from predicted CRISPR arrays, and then searched against reads within the same sample using Blastn [57]. Matches to regions of predicted CRISPRs were discarded, remaining reads were regarded as putative protospacer sources and possible invader sequences. As not all putative protospacers are from invading MGEs (e.g. self targeting spacers), identification of invaders through putative protospacers must be assessed on an ad hoc basis. The subset of putative protospacer reads were then assembled using Canu assembler [49]. Assembled contigs were then annotated utilizing Prokka [58], and circularization of any identified circular genomes were performed using AngularPlasmid [59].

### Datasets

We analyzed two datasets of Illumina TruSeq SLRs. The *gut* dataset has SLRs sampled from the gut microbiome of a healthy human male [43]; the same microbiome was also sequenced using Illumina HiSeq 2000. We downloaded the long reads (SRR2822456) and matching short reads (SRR2822459) of the gut microbiome from NCBI SRA. The other dataset (*mock*) is derived from a synthetic community of 20 organisms with known reference genomes that is widely used for validation [43]. We used its TruSeq SLR dataset (SRR2822457) for comparison purposes: unlike in the gut microbiome, we anticipated to observe no or low dynamics of the CRISPR arrays in the synthetic community of known reference genomes.

We used the long reads directly without assembly for CRISPR-Cas identification. For short reads, we applied MEGAHIT [46] and metaSPAdes [35] to assemble them and then used the contigs to characterize the CRISPR arrays. MEGAHIT [60] and metaSPAdes [61] both utilize an iterative multiple k-mer approach for improving assemblies, and are commonly used assemblers for metagenomes.

### Availability of results and tools

We made available all the results (including the visualization of the compressed spacer graphs) on our supplementary website at http://omics.informatics.indiana.edu/CRISPRone/long. Programs for generating clusters of spacer-sharing CRISPR arrays and for generating a compressed spacer graph from an input file of CRISPR arrays and its visualization (in pdf file using graphviz) can be downloaded from https://github.com/mgtools/crisprlong.

## Data Availability

The programs are available at https://github.com/mgtools/crisprlong. The results are availalbe at http://omics.informatics.indiana.edu/CRISPRone/long.

## Abbreviations

Cas: CRISPR-associated gene
CRISPR: Clustered regularly interspaced short palindromic repeats
MGE: Mobile genetic element
SLR: Synthetic long read
SRA: Sequence read archive
